# Clinical Profile and Outcomes of Epidemic Dropsy Patients Attending a Tertiary Care Centre in Assam, India

**DOI:** 10.7759/cureus.37408

**Published:** 2023-04-10

**Authors:** Tribeni Sharma, Bhaskar Brahma, Afzal Ahmed M Laskar, Joydeep Paul

**Affiliations:** 1 Medicine, Guwahati Medical College and Hospital, Guwahati, IND; 2 Medicine, Tezpur Medical College and Hospital, Tezpur, IND

**Keywords:** erythema, edema, cardiac failure, argemone mexicana oil, epidemic dropsy

## Abstract

Background

The clinical condition of epidemic dropsy is caused by the consumption of edible oils contaminated with Argemone mexicana oil. Two of the most toxic alkaloids found in argemone oil are sanguinarine and dehydrosanguinarine, which cause capillary dilation, proliferation, and increased permeability. Extreme cardiac decompensation leading to congestive heart failure and glaucoma resulting in blindness are the most serious consequences of epidemic dropsy.

Materials and methods

All patients attending the medicine department of Tezpur Medical College and Hospital with clinical features of epidemic dropsy were included in the study after obtaining informed consent. All patients, after a complete history, underwent a thorough clinical examination, and findings were recorded using a pre-formed proforma. Along with routine blood examination, patients were also evaluated with echocardiography, ECG, and chest X-ray. Cooking oil samples obtained from patients were investigated for the presence of sanguinarine in a standardized laboratory with the help of the district authority. The statistical analysis was done using MS Excel 2017.

Results

Out of 38 patients, 36 were male (94.7%), and only two were female (5.2%). Male to female ratio was 18:1. This difference in sex ratio may be due to the fact that only severely ill patients attended our tertiary care hospital. In contrast, moderate and mildly ill patients were treated in local hospitals. The mean age of patients was 28.1 years, and the mean length of hospital stay was eight days. Bilateral pitting type of ankle edema was the most common clinical manifestation, and all 38 patients (100%) exhibited edema. A total of 76% of patients had dermatological manifestations. Sixty-two percent of patients had gastrointestinal manifestations. In cardiovascular manifestation, persistent tachycardia was seen in 52% of patients, pansystolic murmur was best heard in the apical area in 42% of patients, and 21 percent had evidence of a raised jugular venous pressure (JVP). Five percent of patients had pleural effusion. Sixteen percent of patients had ophthalmological manifestations. Eight patients (21%) required ICU care. The in-hospital fatality rate was 10.53% (n=4). Of the expired patients, 100% were male. The most common cause of death was cardiogenic shock (75%), followed by septic shock (25%).

Conclusion

From our study, it was found that most of the patients were male, with an age group of 25-45 years. The most common clinical manifestation was dependent edema, along with signs of heart failure. Other common manifestations were dermatological and gastrointestinal. The severity and outcome were directly related to the delay in seeking medical consultation and diagnosis.

## Introduction

Epidemic dropsy is a clinical condition that occurs when individuals consume edible oils that have been contaminated with Argemone Mexicana oil [1]. Two of Argemone oil's most toxic alkaloids, sanguinarine and dehydrosanguinarine, cause extensive capillary dilatation, proliferation, and enhanced capillary permeability. Oedema occurs when the protein-rich plasma component leaks into the extracellular space [2]. The most disastrous complication of epidemic dropsy is severe cardiac decompensation leading to congestive heart failure and blindness because of glaucoma [3]. Lyon reported the first case of epidemic dropsy from Calcutta, India, in 1877 [4]. West Bengal, Bihar, Orissa, Madhya Pradesh, Uttar Pradesh, Gujarat, Haryana, Maharashtra, Delhi, Jammu and Kashmir, Assam, Rajasthan, and Punjab have all reported cases or outbreaks since that time [5]. The largest outbreak occurred in New Delhi in 1998, resulting in over 3000 hospitalizations and over 60 fatalities [1]. Following the Delhi outbreak, epidemics struck the Indian areas of Gwalior (2000), Kannauj (2002), Lucknow (2005), Panchmahal, and Dungarpur (2012) [6]. Typically, epidemics in India have been reported between July and September, but due to increased law enforcement and public awareness, the mode of presentation has shifted from epidemic to sporadic. Epidemic dropsy is a disease of the old world, and not many cases are reported often. In the month of august 2020, two patients were admitted to the medicine department of Tezpur Medical College and Hospital with complaints of swelling of legs and shortness of breath from adjoining Dhendai tea Estate, Rangapara of Sonitpur district of Assam. Several more cases with similar presentations were admitted in the same week from the same tea estate and the adjacent Borjuli tea estate, where some belonged to the same family. With such a surge in cases from the same area exhibiting bilateral leg swelling and shortness of breath, a probable diagnosis of epidemic dropsy was considered. With collaboration from the district health society edible oil, sampling was done from households of Dhendai and Borjuli tea estates, and the presence of sanguinarine was confirmed, thereby establishing the diagnosis of epidemic dropsy. In the following weeks, 38 patients visited the outpatient department and the emergency department of Tezpur Medical college and hospital with features of epidemic dropsy.

Rationale of the study

Epidemic dropsy is a disease of the old world; the disease occurs in clusters, and the incidence is infrequent. Therefore, it is impertinent to determine the sensitivity and specificity of the clinical features and revisit the natural history and clinical vignette of the disease with special reference to geographic diversity.

## Materials and methods

Our descriptive study was carried out on 38 patients attending the medicine department of Tezpur Medical College and Hospital in North Eastern India, who had symptoms of epidemic dropsy like bilateral lower limb swelling, belonged to the same locality or family, and had a history of consumption of mustard oil contaminated with argemone oil. The duration of the study was one year. Patients below the age of 12 years were excluded from the study. From August 2020 to November 2020, 38 patients were admitted with bilateral lower limb swelling and associated features from Dhendai and Borjuli Tea Estate of the Rangapara area of Sonitpur district, Assam. A thorough and focused history was taken from each patient with special impetus to the disease's onset, progression, and duration. A dietary history with special reference to the type of edible oil consumed was obtained. The study did not include patients with a history of chronic liver disease, congestive heart failure, and chronic kidney disease. All patients underwent mandatory screening for SARS-CoV-2 via both rapid antigen and RT-PCR. Patients with COVID positive status were not included in the study. A comprehensive physical examination was performed on each patient, including an ophthalmological examination that included visual acuity, tonometry, and fundoscopy. Routine blood examinations, including a complete hemogram, liver function test, renal profile, and electrolytes, were also evaluated with electrocardiogram, echocardiography, and chest X-ray. Cooking oil samples were collected from patients' families and sent to the Defence Research Laboratory, Tezpur, Assam, where the presence of sanguinarine was confirmed.

The data collected consisted of demographic profiles of patients, which included sex and age distribution, clinical manifestations including site and extent of edema, gastrointestinal manifestation like vomiting diarrhea, ascites or organomegaly, dermatological manifestations, cardiovascular manifestations, which primarily consisted of persistent tachycardia, pan systolic murmur, raised jugular venous pressure (JVP). All patients' outcomes were also documented in terms of being discharged from the hospital if they expired during the hospital stay. The cause of death of the expired patients was also recorded.

The acquired data were evaluated using Microsoft Excel 2017 and displayed in tables, figures, graphs, and diagrams as appropriate. The findings are reviewed in light of previous research findings with comparable objectives. Since the present study merely examines the frequency distribution of clinical features and outcomes, no significance tests were conducted. 

Ethical clearance was obtained from IRB, Tezpur Medical College and Hospital's ethical committee (approval no. IHC No. 015/TMCH).

## Results

Demographic data

**Figure 1:**
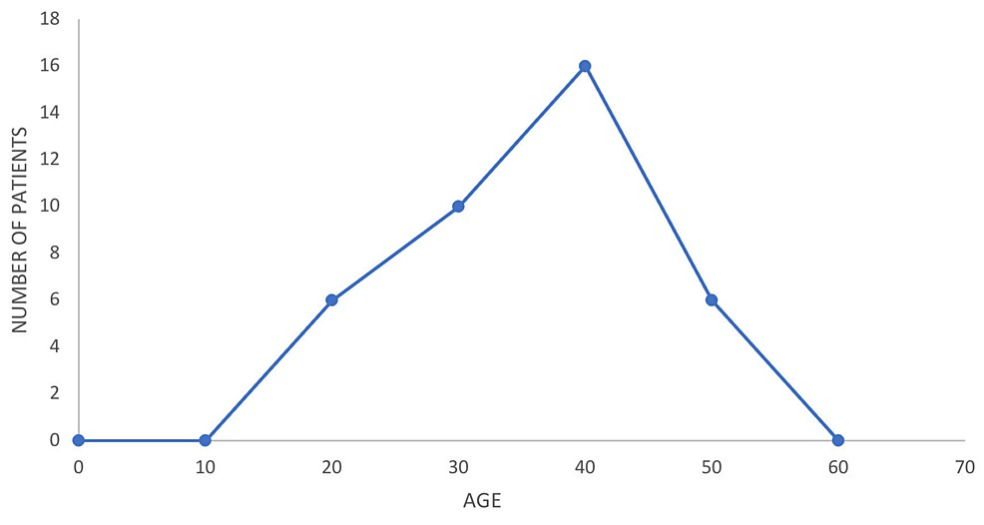
Age distribution. The youngest patient in our study was 14 years and the eldest was 48. The mean age was 28.1 years with a SD of 8.43. The maximum clustering of cases was between the age of 30 and 40 years.

The youngest patient in our study group of epidemic dropsy patients was 14 years, and the eldest was 48. The mean age was 28.1 years. Maximum clustering was seen in the age group of 30-40 years. The age distribution is represented in Figure 1.

**Figure 2:**
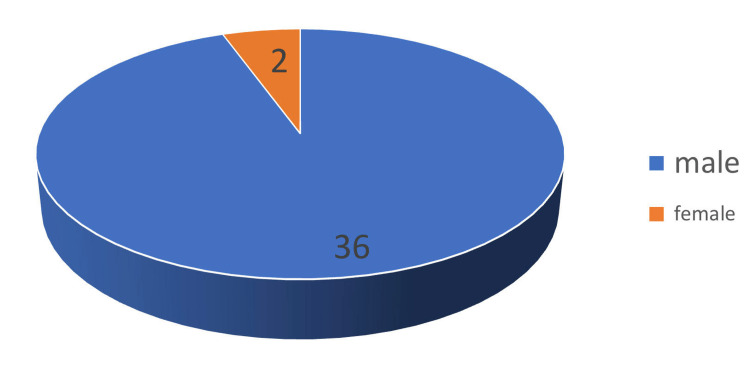
Sex ratio. Out of 38 patients diagnosed with epidemic dropsy, 36 were male and two patients were female. The male-to-female ratio being 18:1.

As represented in Figure 2, the ratio of males to females in our study was 18:1. Out of 38 patients diagnosed with epidemic dropsy, 36 were male, and two were female.

Clinical manifestations

In our study, bilateral pitting edema was the most consistent clinical finding, with 100% (n=38) of patients having this manifestation, as shown in Table 1. A total of 14 patients out of 38 also had upper limb edema. A total of 76% of patients had dermatological manifestations. Among them, 57.89% (n=22) had erythema, 52.63% (n=22) had flushing, and 42.1%(n=16) were developing pigmentation. Fifty percent, that is, 19 out of 38 patients complained of perianal itching. Sixty-two percent of patients had gastrointestinal manifestations, with 55.26% (n=21) having either vomiting, nausea, abdominal pain, or diarrhea. A total of 28.94% (n=11) of patients had hepatomegaly, and splenomegaly was seen in 10.52% of patients (n=4). In cardiovascular manifestations, persistent tachycardia was seen in 52.63% of patients (n=20), pansystolic murmur was best heard in the apical area in 42.1% of patients (n=16), and 21% had evidence of a raised JVP (n=8). A total of 5.26% of patients (n=2) had pleural effusion. Sixteen percent of patients had ophthalmological manifestations in the form of raised intra-ophthalmic pressure (10.52% [n=4]), 5.26% had Roth's spot (n=2), and 5.26% had retinal venous congestion (n=2). 

**Table 1:** Signs and symptoms of patients suffering from epidemic dropsy. JVP: Jugular venous pressure; IOP: Intra-ophthalmic pressure.

Signs and symptoms	Number of patients affected	Percentage
Pitting edema		
Lower limb	38	100
Upper limb	14	36.84
Cutaneous manifestations		
Pigmentation	16	42.1
Tenderness	21	55.26
Erythema	22	57.89
Flushing	20	52.63
Perianal itching	50	19
Gastrointestinal tract		
Nausea, vomiting, abdominal pain, diarrhea	21	55.26
Hepatomegaly	11	28.94
Splenomegaly	4	10.52
Ascites	12	31.57
Cardiovascular system		
Persistent tachycardia	20	52.63
Pansystolic murmur	16	42.1
Raised JVP	8	21
Respiratory system		
Pleural effusion	2	5.26
Ophthalmic manifestations		
Raised IOP	4	10.52
Roth’s spots	2	5.26
Retinal venous congestion	2	5.26

Outcome

**Figure 3:**
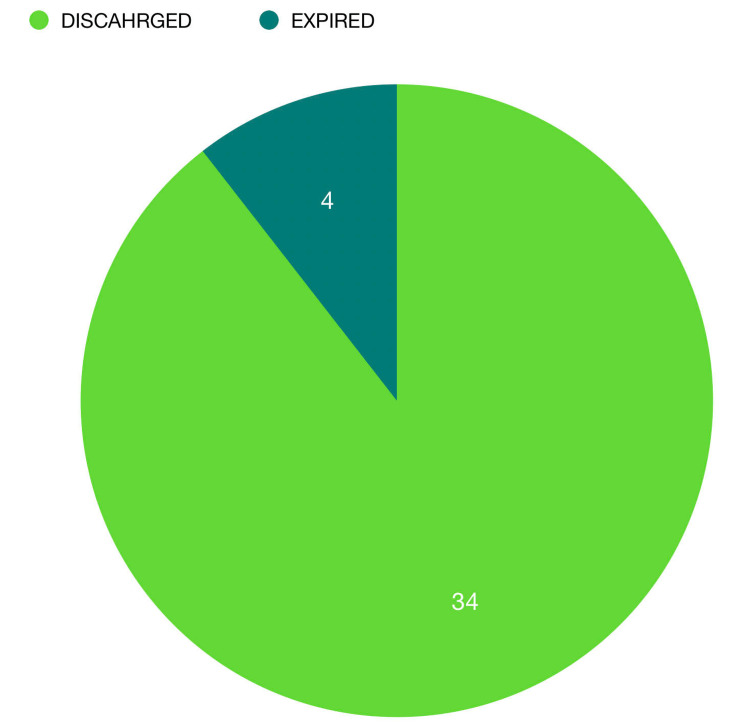
Outcome. Out of the 38 patients, 89.47% (n=34) were discharged, and 10.53% of patients (n=4) expired during their hospital stay.

**Figure 4:**
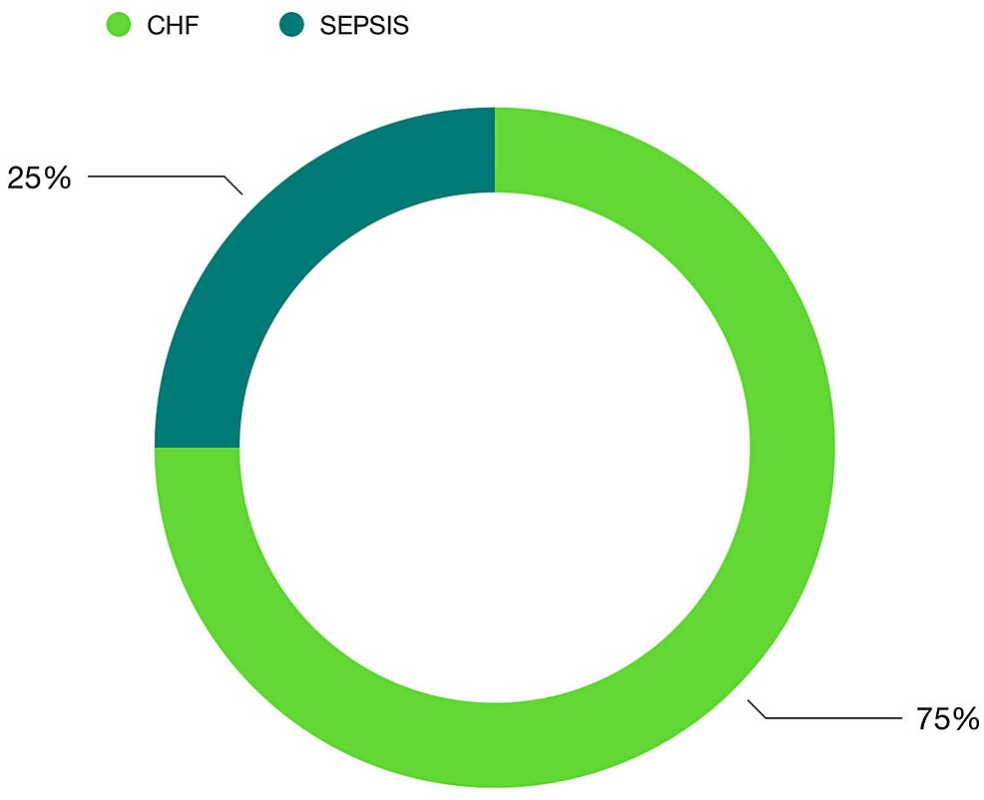
Causes of death. Congestive heart failure was the cause of death in 75% of patients (n=3) with sepsis as the cause in 25% (n=1).

The outcome of the 38 patients is that 89% (n=34) were discharged, 11% of patients (n=4) expired, and congestive heart failure was the cause of death in 75% of patients (n=3) with sepsis as the cause in 25% (n=1) (Figures 3-4).

## Discussion

The diagnosis of epidemic dropsy is based on two criteria: certain common epidemiological characteristics, a cluster of signs and symptoms, and the presence of sanguinarine in the cooking medium. In the current study, an outbreak of epidemic dropsy was established by noting the simultaneous appearance of symptoms in multiple family members, seasonal inclination, typical clinical expression, and the presence of sanguinarine in mustard oil taken from families of affected patients. The seeds of Argemone mexicana contain the toxins sanguinarine and dihydrosanguinarine. In epidemic dropsy, the toxic alkaloid sanguinarine affects the oxidation of pyruvic acid, which accumulates in the blood and promotes capillary dilatation and increased permeability, the predominant clinical symptom of which is widespread edema [1,3,7]. In our study of 38 patients presenting with epidemic dropsy admitted to the medicine department of Tezpur Medical College, the mean age distribution was 28.1 years, with the youngest being 14 years and the oldest age recorded as 48 years. The maximum clustering of patients was between 30 and 40 years old. Azazh A et al. [8] and Sharma BD et al. [1], in their study, found persons of all ages being affected, except breastfed infants and toddlers who have no mustard oil in their diets. Our study excluded the pediatric population. Among the 38 patients, gender distribution was highly skewed, with 36 male and only two female patients. Lal SB [9], in his study titled "Epidemic dropsy in Bihar," found both sexes were affected equally. Tomar LR et al. [6] also found almost equal gender distribution in their study. Since only those patients who needed special attention were admitted to our center and most of the patients were managed at peripheral hospitals, the gender distribution of our study might not reflect the true demographic pattern of the disease. Edema was the most common clinical feature and one of the most important diagnostic criteria. All patients had lower limb edema; however, 36% of patients had upper limb edema as well. This finding is in resonance with previous studies, which also found similar findings [1,2,3]. Edema in epidemic dropsy is due to increased capillary leak, vasodilation, and salt and water retention. The edema was found to be poorly responsive to diuretics. The edema persisted for around six weeks without any specific intervention other than abstinence from contaminated cooking oil, and it resolved spontaneously. However, those patients who developed congestive heart failure required diuretics. Chaudhari RN and Chakravarty NK, in their study also reported that the edema was not responsive to diuretics [10].

The cutaneous manifestation was seen in 76% of patients in the form of erythema, tenderness, and hyperpigmentation. Gomber S et al. [11] in their study also had comparable findings (Table 2). In our study, 50% of patients reported perianal itching, which was not reported in earlier studies from India. Perianal itching did not require any specific therapy, and cessation of exposure resolved the itching. Perianal itching was reported by Singh R et al. [3] in their study conducted in Nepal.

**Table 2:** Comparison of the present study. JVP: Jugular venous pressure.

Clinical features	Gomber S et al. [11]	Present study
Edematous legs	100	100
Local tenderness	70	55.26
Erythema	43.3	57.89
Pigmentation/Skin darkening	46.6	42.1
Perianal Itching	Not reported	50
Cardiovascular system		
Tachycardia	100	52.63
Systolic murmur	13.3	42.1
Raised JVP	6.6	21
Fundus	Not reported	5.2

The cardiovascular manifestation was seen in 52% of patients, mostly mild in the form of persistent tachycardia. However, features of congestive heart failure were seen in 21% of patients with raised JVP, bi-basilar crepitations, and orthopnoea. These findings are in concurrence with previous studies [2,3]. Raised IOP was seen in four patients out of 38, and Roth's spot was seen in two patients along with retinal venous congestion. Singh R et al., in their study, found that 5.8% of patients had raised IOP, whereas retinal venous congestion was reported in 11.76% of patients [3].

Limitation

The sample size of our study is small. Only patients who presented to our hospital with features of epidemic dropsy were included in the study. Our study did not include patients of the pediatric age group, and because our hospital is a tertiary care facility, only critically ill patients were present. A substantial number of patients with similar clinical manifestations were treated in hospitals on the periphery but were excluded from the study. Therefore, our study's demographic distribution may not accurately reflect the outbreak.

## Conclusions

The contamination of mustard oil with Argemone mexicana continues to be the cause of epidemic dropsy. In India, the mustard harvest typically occurs between June and July; consequently, most epidemic dropsy cases occur in August, September, and October. Especially in endemic areas, a high index of suspicion is required when patients from the same family or locality exhibit dropsy symptoms. Edema with diverse cutaneous manifestations is the most common form of presentation. Edema in epidemic dropsy responds poorly to diuretics; however, edema subsides gradually without specific intervention once the exposure has been terminated. A small number of patients did develop symptoms of congestive heart failure, necessitating the use of diuretics and intensive care. The key to preventing symptoms of florid heart failure is termination of exposure to sanguinarine and early diagnosis.
